# The Use of Complementary and Alternative Medicine among Lebanese Adults: Results from a National Survey

**DOI:** 10.1155/2015/682397

**Published:** 2015-05-27

**Authors:** F. Naja, M. Alameddine, L. Itani, H. Shoaib, D. Hariri, S. Talhouk

**Affiliations:** ^1^Department of Nutrition and Food Sciences, Faculty of Agriculture and Food Sciences, American University of Beirut, Riad El-Solh Square, Beirut 1107 2020, Lebanon; ^2^Department of Health Management and Policy, Faculty of Health Sciences, American University of Beirut, Riad El-Solh Square, Beirut 1107 2020, Lebanon; ^3^Department of Nutrition and Dietetics, Faculty of Health Sciences, Beirut Arab University, Riad El-Solh Square, P.O. Box 11-5020, Beirut 1107 2809, Lebanon

## Abstract

*Objective*. To examine the prevalence and correlates of Complementary and Alternative Medicine (CAM) use in Lebanon. *Methods*. A cross-sectional survey was conducted through face to face interviews on a nationally representative sample of 1,475 Lebanese adults. The survey questionnaire explored the sociodemographic and health related characteristics as well as the types and modes of CAM use. The main outcome in this study was the use of CAM during the last 12 months. *Results*. Prevalence of CAM use was 29.87% with “folk herbs” being the most commonly used (75%). Two out of five CAM users indicated using it as alternative to conventional therapies and only 28.4% of users disclosed the use of CAM to their physician. CAM use was significantly associated with higher income, presence of a chronic disease, and lack of access to needed health care. Lower odds of CAM use were observed among older adults and those with a higher education level. *Conclusions*. This study revealed a high prevalence of CAM use in Lebanon. Health policy and decision makers need to facilitate proper regulation and integration of CAM into mainstream medicine and educate health care providers and the public alike on the safe and effective use of CAM therapies.

## 1. Introduction

Complementary and Alternative Medicine (CAM) is a diverse group of medical and healthcare systems, practices, and products that are not considered part of conventional medicine yet complement it by diversifying the conceptual frameworks of medicine or by satisfying a demand not met by orthodoxy [1]. The United States (US) National Center for CAM therapies divides CAM into four categories: mind-body systems, manipulative and body-based practices, energy medicine, and* biologically based practices* [2]. In this paper, CAM refers to biologically based practices including substances found in nature, such as herbs, dietary supplements, multivitamin, and mineral supplements. Medical use of CAM products is on the increase worldwide [3]. The growing popularity of biologically based CAM products has been attributed to a variety of factors including dissatisfaction with conventional medical practices [4], frustration with the contradictory and consistently evolving state of current medical knowledge [5, 6], the increasing cost of conventional medical care [7], the intellectual and spiritual appeal of holistic models of health and healing [8], and the placebo effect [8, 9].

Worldwide, there exists a wide geographical variation in the prevalence estimates of CAM products' utilization and the types of CAM therapies most commonly used. For example, according to the US 2007 Health Interview Survey, 40% of adults used CAM therapy in the previous year. Most common therapies used included natural products and deep breathing exercises [10]. Higher prevalence of use is reported among older Ontarians. More specifically, 51% reported using one or more natural health product during the previous 12 months [11]. In England, 26% of respondents used CAM in the past 12 months; the most commonly used were massage, aromatherapy, and acupuncture [12]. A study on CAM use in Taiwan reported that 38% of respondents used CAM at least once during the previous 12 months, mainly in the form of medicinal herbs and health supplements [13]. A study on dietary supplements by South Korean adults showed that about 62% of adults had taken a dietary supplement during the previous 12 months [14]. Prevalence of biologically based therapies use among adults in Nigeria was estimated to be 47% [15].

High prevalence of CAM use is also reported in the Arabian Gulf Region. For example, a National study in Saudi Arabia revealed that 74% of study respondents visited a CAM provider in the previous 12 months, including spiritual healers, herbalists, honeybee providers, and hijama (Arabic name for wet cupping) [16]. A comparable prevalence of CAM use (71%) was also reported in Kuwait, and the main type of CAM used was herbal remedy [17].

The popularity and increased use of CAM products have led to concerns about their safety and possible health risks. Health problems from CAM products' use have been reported in the lay media, by government agencies, and by published studies in medical journals [18, 19]. Such health problems can arise from improper use of the product, improper/inaccurate content, and product tampering and defects that can alter product identity, quality, purity, strength, and/or composition [20]. In addition, certain CAM modalities may cause adverse reactions such as causing interactions with conventional medicines, worsening the side effects of conventional therapies, or negatively influencing compliance to conventional treatment compromising its desired outcome [21, 22].

Historical and current studies indicate that the Eastern region of the Mediterranean (EMR), which includes most Arab countries, has been distinguished from other regions by a rich inventory of CAM products, in particular, herbal medicine [23]. However, published research on the prevalence of use and other associated factors in this region is scarce. A recent study in Lebanon examined the regulation of CAM products, including herb and dietary supplements, and concluded that existing regulatory frameworks and mechanisms are not conducive to public safety and do not support the proper integration of CAM products into the healthcare system [24]. Investigating the prevalence, characteristics, outcomes, and determinants of CAM use is thus of great importance to policy makers as it would inform the formulation of policies and procedures that would enhance the safe and effective use of CAM products and would assist in the integration of CAM therapies into mainstream medicine [12].

The main objectives of this study were to estimate prevalence of CAM products use in a nationally representative sample of Lebanese adults and to identify specific factors associated with such use. In addition, this study aimed at characterizing the patterns and underlying causes as well as perceived efficacy of CAM use.

## 2. Methods

The study protocol was approved by the research ethics board (Institutional Review Board) at the American University of Beirut, Protocol number FHS.MA.03.

### 2.1. Study Design and Data Collection

A cross-sectional survey was conducted on a nationally representative sample of 1,475 Lebanese adults in years 2010 and 2011. Sampling was performed using a multistage Probability Proportional to Size (PPS) Cluster Sampling. The survey sample was allocated between the governorates of Lebanon (Beirut, Mount Lebanon, North Lebanon, South Lebanon (including Nabatiye), and Bekaa). National quotas for age and gender were also allocated. Subjects were recruited in each quota until it was filled. In the absence of national census or household rosters in Lebanon (due to unwarranted political reasons), the reference population numbers used were provided by the National Survey of Household Living Conditions conducted by the Central Administration for Statistics in Lebanon in 2004 [25]. Out of 10,651 clusters, a total of 160 were randomly chosen as primary sampling unit in this survey. Each cluster is formed of 100 to 150 households. Random sampling was also used to select 8–10 households from each cluster. All adults over the age of 18 years who were available at the time of interview in the household were listed on a Kish table matrix and one adult was selected at random.

Participation was voluntary, and informed consent was obtained from all subjects prior to participation. No financial incentive was offered. The average administration time of the interviews was 20 minutes. Sample size calculations showed that 1475 subjects are needed to provide 95% confidence interval around a CAM use prevalence estimate of 40% with ±2.5% variation.

### 2.2. The Survey Questionnaire

For the purpose of this survey, CAM referred to “biologically based practices” and included dietary supplements, herbal products, and the other so-called natural but yet scientifically unproven therapies [2]. In face to face interviews, respondents completed a multicomponent questionnaire. The latter was developed by the research team and thoroughly reviewed by an expert panel consisting of a medical doctor, an epidemiologist, a health management and policy expert, and an economist. The questionnaire included questions regarding personal use of CAM during the previous 12 months and the type of CAM product used. The questionnaire also investigated the following characteristics of the CAM product use: frequency, reason of use, motives that led to the use of CAM, main source of advice about the CAM use, mean out of pocket expenditure on CAM products during the previous 12 months, satisfaction with CAM use, and disclosure of CAM use to an individual's physician as well as the attitude toward CAM use. The questionnaire further explored the sociodemographic and health related characteristics of respondents, including age, sex, education (categorized as primary education or lower, high school or diploma degree, and university degree or higher), marital status (married, unmarried), employment status (formal employment, self-employed, and unemployed), having a chronic disease (yes, no), and whether respondents felt they needed health care but did not receive it (yes, no). In addition, the crowding index, a composite variable composed of the number of household members as the numerator and the number of rooms used for sleeping as the denominator, was assessed. Several epidemiological studies have correlated a high household crowding index with low socioeconomic status [26, 27]. Anthropometric measurements including weight and height were taken using standardized techniques and calibrated equipment. Body mass index (BMI) was calculated as the ratio of weight (kilograms) to square of height (meters). The final survey questionnaire was piloted on 35 randomly selected individuals who were asked to provide feedback on the clarity and flow of the questionnaire. All feedback received was incorporated in the final version of the questionnaire.

### 2.3. Statistical Analysis

Descriptive statistics were calculated as means, percentages, or absolute figures when applicable. Demographic and clinical characteristics between CAM users and nonusers were compared. Differences in proportions were tested using chi-square test, and means were compared by means of Student's *t*-test. Multivariate logistic regression analysis was utilized to identify the characteristics significantly associated with CAM use. To determine the predictors of CAM use, variables that met significance in the univariate logistics regression analysis were entered in the final multivariate model. Odds ratios and 95% confidence intervals were shown. All analyses were performed using SPSS 18 [28].

## 3. Results

Out of 2,356 adults approached, 1,500 agreed to participate in this survey (63.7% response rate). The main reported reasons for refusal to participate were lack of time (46.4%) and lack of interest in the study (25.8%). The distribution of the sample population obtained was proportional to that of the general Lebanese population with respect to governorate, age, and sex.

### 3.1. Prevalence and Types of CAM Products Used

Out of the 1,500 survey respondents, 448 reported using a type of CAM during the previous 12 months (29.9%, 95% CI: 27.61–32.24). Upon further analysis, CAM products used were grouped into “Folk Herbs,” “Folk Foods,” “Natural Health Products,” and “Vitamins and Minerals” supplements. Below are further details on each group:

- *Folk Herbs* included all infusions made with traditional herbs and herbal extracts which were used to treat and/or prevent a health condition. Examples of such infusions were green tea, Anise tea, Thyme tea, and a mixture of specific flowers believed to prevent or treat common cold symptoms locally referred to as “Zhourat.”
- *Natural Health Products* are products other than Vitamins and Minerals that presented a health claim. This category includes natural products and mixes with claims of treatment of multiple health conditions including obesity, chronic diseases (such as diabetes, hypertension, high blood cholesterol, and triglycerides), and energy boosters.
- *Folk Foods* referred to functional foods that were included in the diet for their believed health benefits, like honey, black seed, molasses, artichoke, onion, garlic, and asparagus.
- *Vitamins and Minerals* consisted of single, multimineral, multivitamin, and multivitamins and mineral products.

Analysis of survey data reveals that the most popular forms of CAM products used were “Folk Herbs” (75%), “Natural Health Products” (31.7%), “Folk Foods” (13.2%), and “Vitamins and Minerals” supplements (3.8%) (Table 1). Among the herbs, green tea, Chamomile, Anise, and Peppermint were the most commonly used. Mixtures of natural herbs packed and marketed by local companies were the mostly used Natural Health Products (90% of products used). Honey and Molasses constituted over 50% of total “Folk Foods” consumed. Out of all the Vitamins and Minerals used, vitamin C was the most commonly used supplement (20%).

### 3.2. CAM Use Characteristics

Table 1 exhibits the characteristics of CAM use among CAM product users during the previous 12 months (*n* = 448). Daily use was limited to 26% of CAM users. The main reasons for using the CAM product were for treatment and prevention of flu and common cold (30.3%), indigestion and stomach problems (25.4%), and slimming and weight loss (11%). As for the motives that led to the use of a certain CAM product, “belief in the advantages of CAM products” was reported by 76.3% of CAM users. Other motives included “trying because of a suggestion” (12.6%), feeling of having no alternative (8.3%), and “disappointment with conventional medical therapy” (7.4%). When asked about how they were informed about the CAM product they have used, 58.3% of subjects said that it was passed to them through family traditions, 24.8% of them reported it as a personal choice, 13.8% cited their friends as the main source of information, and only 5.8% were told about the CAM product by a health care practitioner. Most of the CAM users were satisfied with the CAM product used (90%) and were to advise others to use the same product (89.4%). Among the dissatisfied CAM users, the main reason for dissatisfaction was lack of improvement or benefits (81.8%) and the experience of negative side effects (18.2%). “Dissatisfaction with the CAM product used” was further investigated in relation to the type of CAM. Results indicated that the highest rates of CAM user dissatisfaction were reported among users of “Vitamins and Minerals” (24.5%) and “Natural Health Products” (12.7%). The lowest rates of dissatisfaction were observed among users of “Folk Herbs” (3.8%) and “Folk Foods” (8.5%) (data not shown).

The average monthly household spending on CAM was 19,000 ± 32,000 Lebanese pounds, which is equivalent to 12.7 ± 21.3 United States dollars (USD). Taking into consideration that the total number of households in Lebanon is estimated at 752,000 [25], the total national household spending would range from USD $9.55 to USD $25.57 million. Note that the vast majority of this spending is out of pocket since CAM products are neither covered by public/social insurance nor subsidized by private insurance.

Sixty percent of CAM users indicated they utilized the CAM product as complementary to conventional medicine while 40% used it as alternative. CAM use was mostly irregular (53%). Seventy-two percent of CAM users did not disclose their CAM use to their physician, and when asked about the reasons for not telling the physician, 58.3% of respondents felt there was no need to so, 15.2% thought that doctors do not believe in CAM, and 10.5% were convinced that a CAM product can do no harm. Among subjects who informed their physician about the CAM use, 79% said that the physician encouraged the use, 12.9% said the provider discouraged it, and 8% reported that the physician was neutral.

### 3.3. Comparing CAM Users to Nonusers

Sociodemographic and health related characteristics of CAM users and nonusers are presented in Table 2. Subjects younger than 30 and older than 50 had lower odds of using CAM as compared to those between 30 and 50 years of age (OR: 0.78, 95%: 0.6–0.96; OR: 0.70, 95% CI: 0.52–0.93, resp.). Females had significantly higher odds of CAM use (OR: 1.18, 95% CI: 1.01–1.46). As for education, the percent of CAM users decreased with increasing level of education with the odds of CAM use among subjects with university degree being significantly lower compared to that among subjects with elementary or lower level of education (OR: 0.63, 95% CI: 0.45–0.89). In contrast to education, percentage of CAM users increased with increasing income, with subjects earning more than USD $2,000 per month being 1.5 times more likely to use a form of CAM as compared to those who earn less than USD $1000 (OR: 1.5, 95% CI: 1.1–2.03).

CAM use was more frequent among subjects with a chronic disease (OR: 1.5, 95% CI: 1.14–1.91) and among those who felt that they needed health care but did not receive it during the previous 12 months (OR: 2.36, 95% CI: 1.81–3.07). After adjustment for other variables using multivariate logistic regression, age, education, income, presence of a chronic disease, and unmet health need were significantly correlated with CAM use (Table 3).

## 4. Discussion

This study is the first national survey on the prevalence, correlates, and characteristics of CAM use among Lebanese adults. Close to one in three Lebanese adults reported using a type of CAM products in the previous 12 months, with “Folk Herbs” contributing three-quarters of such use. The vast majority of CAM users were satisfied with their utilization of CAM products. CAM use was higher among middle aged individuals, females, patients with chronic diseases, and those reporting unmet health needs. The prevalence of CAM use increased with the increase in household income and decreased with the level of education of CAM users. Most CAM users did not disclose their CAM use to their physician.

A main finding in this study is the prevalent use of traditional CAM therapies, especially “Folk Herbs” and “Folk Foods,” with an overwhelming satisfaction with such use. This could be justified by the deep rooted historical, cultural, and religious underpinning for CAM use in Lebanon and other Arab countries. Historically, the use of traditional CAM products was at the core of traditional Arab medicine. Family traditions have transmitted ancestral knowledge of a region earlier referred to as Bilad al Sham, the Levant, that was endowed with a high floral and herbal diversity all around the year. Such diversity constituted a basis for health care in the region with very few species imported from outside [29, 30].

More than three-quarters of survey respondents in this study believe that herbal medicine and foods are beneficial and that their use is part of the family's traditional way of enhancing the well-being of the body. Sociocultural traditions coupled with family heritage have managed to maintain relics of this historical tradition alive into the 21st century. Currently, fewer than 200–250 plant species are still in use in Arab tradition medicine for the treatment of various diseases [30]. Arab families still include in their repertoire of medicinal use many of these plants even though very few have had their medicinal properties investigated using contemporary evidence based medicine [31, 32].

This study further reveals that two out of five users utilize CAM products, especially Folk Herbs, on an alternative basis. This is mainly due to the public belief that the use of such therapies to treat common and less serious medical conditions (common cold, stomach discomfort) is safe with no side effects. More importantly, the cultural value of traditional Arab medicine remains part of the collective memory of the people of the region despite its marginalization and decline and most studies show that people in the Arab world still rely on traditional medicine for health care either as primary care [31, 33] or as complementary care [33–35].

Yet, this rich and resilient cultural heritage for Folk Herbs in Lebanon might be threatened by mostly imported CAM therapies, including Natural Health Products and Vitamins and Minerals, due to the absence of scientific evidence for their use coupled with poor regulatory frameworks [24]. Although the majority of CAM users expressed satisfaction with CAM use and willingness to recommend such use to others, nonsatisfied users indicated concerns with lack of any improvement or even more seriously with reported side effects. This is important since the use of CAM therapies in Lebanon is not exclusive to the treatment of common and less serious conditions. Previous studies reveal that CAM therapies may be used, albeit at a lower prevalence, to treat more chronic and difficult to treat conditions such as infertility (40% prevalence) [35] and pediatric leukemia (15.2% prevalence) [36]. Such use may be coupled with concerns regarding professional ethics or know-how in relation to quality, efficacy, and safety of the natural formulations. Concerns are attributed to adulteration, inappropriate formulation, or lack of understanding of plant and drug interactions or uses which may lead to adverse reactions in patients [37].

Unfortunately, the popularity of CAM products in Lebanon has not been coupled with educational and regulatory mechanisms to better integrate those products into mainstream medicine [24]. Such an issue is common in other Arab countries as traditional Arab medicine in general and traditional CAM therapies in particular have failed to integrate into mainstream medicine [30]. Concerned stakeholders and decision and policy makers are invited to reflect on the findings on this study in order to enhance the regulation and integration of CAM therapies, especially the very popular herbal remedies, into mainstream medicine.

Study results reveal a number of factors that increase the propensity of CAM use, including female gender, middle age, presence of chronic diseases, and unmet health needs. The finding that females use CAM more frequently than males concurs with international literature and national surveys that showed that the propensity of CAM use was higher among females [12, 17, 38, 39]. This could be attributed to a plethora of factors including females' use of CAM therapies for the treatment of menstrual pains or for weight reduction [40–42]. The finding regarding a higher probability of CAM use among middle aged individuals is also in agreement with previously reported studies [43]. Study analyses further reveal that the prevalence of CAM use increased with the increase in household income, a finding well supported in literature [44–47]. The findings of this study are also in agreement with previous studies in regard to the higher probability of CAM use among patients with unmet health needs and those suffering from chronic or life threatening illnesses [12, 16, 36, 48]. From a practice perspective, pre- and postservice care providers need to be empowered with the knowledge and skills needed to discuss with their patients the safe use of CAM therapies with a particular focus on females and on patients with chronic diseases and serious illnesses. In regard to preservice capacity building, medical, nursing, and health curricula need to be revised in order to educate and train students on the means to properly advise their patients on proper use of CAM therapies. Continuing education and professional development programs need to be organized for postservice providers in order to enhance their knowledge and skills on evidence based use of CAM therapies. The orders and syndicates could possibly play a major role with such training.

One disconcerting finding in this study relates to the use of CAM remaining overwhelmingly undisclosed to physicians. Such a finding is in accordance with studies carried out in other countries/contexts [39, 41, 49, 50]. Multiple reasons have been expressed for this lack of disclosure, including not wanting to visit a physician, fear that physicians would discourage CAM use, belief that CAM use needs not to be discussed with providers, and because CAM use does not hurt anyway. Such reasons are supported by literature [51, 52]. However, of the minority that did inform their physicians about the use of CAM, four out of five reported that the physicians were supportive of such use. Such findings flag the need for the Ministry of Health, health care institutions, orders, and syndicates to work collaboratively on educating care providers on discussing CAM use with their patients. Education institutions should also work on integrating CAM into medical and nursing curricula. Finally, there is a need to partner with media outlets in delivering awareness campaigns that encourage the public on the safe use of CAM.

There are a number of limitations that are worthy of mention in this study. First, it cannot be ascertained whether nonrespondents to the household survey would reveal a different prevalence or pattern of use of CAM therapies as compared to respondents. The reasons provided of nonrespondents may in fact conceal absence of or dissatisfaction with CAM use. Second, since respondents were asked to report CAM use over the last twelve months, recall bias may be a risk especially for those that use CAM sparsely and irregularly. Third, despite every attempt by the research team to define the meaning and types of CAM to respondents, it cannot be assured that all respondents were well aware of all types of CAM that could potentially be used, which may have resulted in underestimating such use in the current study. Fourth, the cross-sectional design in this study does not allow for establishing causality but rather significant associations between CAM use and other variables of interest.

This study revealed a high prevalence of CAM use in Lebanon, particularly for herbal remedies and Folk Foods. Such use has been enshrined in cultural practices and family traditions for hundreds of years and as such is expected to remain and perhaps flourish in the future with the rise in chronic and serious illnesses. Health policy and decision makers should dedicate concerted efforts on facilitating the proper regulation and safe integration of CAM therapies into mainstream medicine and on educating health care providers and the public alike on the safe and effective use of CAM therapies.

## Figures and Tables

**Table 1:** Characteristics of CAM use among study respondents who reported using CAM product during the previous 12 months (*n* = 448).

Characteristics of CAM use	*n* (%)
*Types of CAM used * ^*∗*^	
Folk Herbs	336 (75)
Natural Health Products	142 (31.7)
Folk Foods	59 (13.2)
Vitamins and Minerals	17 (3.8)

*How often do you use the product? (n* = *448) *	
Irregular use	238 (53.1)
Daily	116 (25.9)
More than once per week	69 (15.4)
Weekly	25 (5.6)

*Why have you used CAM? (n* = *433) * ^*∗*^	
Flu	131 (30.3)
Digestion and stomach problems	110 (25.4)
To enhance energy	87 (20.1)
Enhancing immunity	78 (18.0)
Treatment of a chronic illness	54 (12.5)
Other	53 (12.2)
For slimming and weight loss	47 (10.9)

*What are the motives which led to use of CAM?* *(n* = *435) * ^*∗*^	
Belief in advantages of complementary and alternative medicine practices	332 (76.3)
Trying because of a suggestion	55 (12.6)
Feeling of having no alternative	36 (8.3)
Disappointment from conventional medical therapy	32 (7.4)
Family traditions	17 (3.9)
Other	13 (3)

*How did you hear about CAM? (n* = *434) * ^*∗*^	
Family tradition	253 (58.3)
Personal choice	108 (24.9)
Friends	60 (13.8)
Media	56 (12.9)
Health practitioner	25 (5.8)
Neighbors	17 (3.9)
Books	11 (2.5)

*Were you satisfied with the CAM product you used? (n* = *428) *	
Yes	385 (90.0)
No	43 (10.0)

*If no, why? (n* = *33) *	
There was no improvement or benefits	27 (81.8)
Caused side effects	6 (18.2)

*Do you advise others to use the same CAM product? (n* = *424) *	
Yes	379 (89.4)
No	45 (10.6)

*Complementary or alternative to conventional medicine (n* = *187) *	
Complementary	246 (60.1%)
Alternative	163 (39.9%)

*Have you asked your doctor about the CAM product you used? (n* = *437) *	
Yes	124 (28.4)
No	313 (71.6)

*If no, why? (n* = *276) *	
Do not like to go to doctors	161 (58.3)
CAM does not hurt	42 (15.2)
Doctors do not believe in CAM	29 (10.5)
There was no need	26 (9.4)
Other	18 (6.5)

*How was the reaction of the physician? (n* = *124) *	
Encouraging	98 (79.0)
Not encouraging	16 (12.9)
Neutral	10 (8.1)

Average monthly household spending on CAM in Lebanese lira* (n* = *438) * ^*∗∗*^	19,000 ± 32,000

^*∗*^Percentages do not add to 100% because more than one answer was reported.

^*∗∗*^1 US$ is equivalent to 1,500 Lebanese liras.

**Table 2:** Prevalence and odds of CAM use according to various sociodemographic, lifestyle, and health characteristics in a nationally representative sample of Lebanese adults (*n* = 1500).

Sociodemographic and health related characteristics	Overall *n* (%)	Prevalence of CAM use (%)	OR (95% CI)
*Age *			
31–50 years	588 (39.2)	33.7	1.00
≤30 years	543 (36.2)	28.2	**0.773 (0.600–0.996)**
>50 years	369 (24.6)	26.3	**0.702 (0.527–0.937)**

*Sex *			
Males	816 (54.4)	28.3	1.00
Females	684 (45.6)	31.7	**1.177 (1.01–1.469)**

*Education *			
Primary education or lower	337 (22.5)	33.2	1.00
High school or technical school	827 (55.1)	30.8	0.896 (0.683–1.174)
Higher education (university degree)	336 (22.4)	24.1	**0.638 (0.455–0.894)**

*Income *			
<1000	841 (56.1)	28.4	1
1000–2000	428 (28.5)	28.7	1.016 (0.785–1.314)
>2000	231 (15.4)	37.2	**1.494 (1.100–2.029)**

*Marital *			
Unmarried	600 (40)	28.5	1.00
Married	900 (60)	30.8	1.115 (0.889–1.399)

*Employment *			
Unemployed	464 (30.9)	30.0	1.00
Employed	1036 (69.1)	29.8	0.994 (0.782–1.262)

*Do you have a chronic disease? *			
No	1171 (78.1)	28.0	1.00
Yes	329 (21.9)	36.5	**1.476 (1.140–1.910)**

*During the last year, did you feel that you needed health care but did not receive it? *			
No	1217 (81.1)	26.2	1.00
Yes	283 (18.9)	45.6	**2.358 (1.806–3.076)**

*Body mass index (Kg/m* ^2^)			
BMI < 25	690 (46.0)	30.3	1.00
25 ≤ BMI < 30	548 (36.6)	27.9	0.981 (0.696–1.142)
BMI ≥ 30	261 (17.4)	32.6	1.111 (0.819–1.509)

**Table 3:** Determinants of CAM use using multivariate logistic regression.

Sociodemographic and health related characteristics	CAM use
	OR (95% CI)
*Age *	
31–50 years	1.00
≤30 years	0.93 (0.71–1.21)
>50 years	**0.64 (0.46–0.89)**

*Sex *	
Males	1.00
Females	1.17 (0.92–1.47)

*Education *	
Primary education or lower	1.00
High school or technical school	0.86 (0.64–1.16)
Higher education (university degree)	**0.58 (0.4–0.85)**

*Income *	
<1000	1
1000–2000	1.24 (0.95–1.63)
>2000	**2.18 (1.56–3.06)**

*Do you have a chronic disease? *	
No	1.00
Yes	**1.41 (1.04–1.91)**

*During the last year, did you feel that you needed health care but did not receive it? *	
No	1.00
Yes	**2.27 (1.7–3.03)**
